# A GM-CSF-Flt3L Fused Adjuvant Enhances Immune Responses and Protective Efficacy of Influenza DNA Vaccine in Mice

**DOI:** 10.3390/vaccines14080671

**Published:** 2026-08-02

**Authors:** Hongzhe Lin, Mingyue Chen, Rong Xiang, Yating Kang, Yiwei Zhong, Duan Ma, Bin Wang

**Affiliations:** 1 Key Laboratory of Medical Molecular Virology (MOE/NHC/CAMS) & Department of Biochemistry and Molecular Biology, School of Basic Medical Sciences, Fudan University, Shanghai 200032, China; 2 Shanghai Institute of Infectious Disease and Biosecurity, Shanghai 200032, China; 3 Advaccine Biopharmaceuticals Suzhou Co., Ltd., Suzhou 215123, China

**Keywords:** DNA vaccine, GM-CSF-Flt3L adjuvant, GM-CSF adjuvant, Flt3L adjuvant, influenza

## Abstract

Background: DNA vaccines are widely used due to their low production cost, ease of large-scale manufacturing, and rapid responsiveness to emerging epidemics. However, their relatively modest immunogenicity in larger species often requires potent adjuvants to elicit robust protective responses. GM-CSF and Flt3L are two cytokine adjuvants targeting dendritic cells (DCs). Although their combination has been reported to augment immune responses, the mechanism underlying the complementary effects of a genetically fused GM-CSF-Flt3L adjuvant plasmid remains incompletely understood. Methods: We constructed a GM-CSF-Flt3L fusion adjuvant plasmid and compared it with single-adjuvant plasmids for their effects on innate, humoral, and cellular immunity, as well as protective efficacy against lethal homologous virus challenge. Results: Compared with single-adjuvant plasmids, GM-CSF-Flt3L synergistically promoted the maturation of bone marrow-derived dendritic cells (BMDCs) and activated draining lymph node DCs, differentially expanded DC subsets, and enhanced the recruitment of migratory DCs. This adjuvant significantly elevated hemagglutinin (HA)-specific serum IgG titers, hemagglutination inhibition (HI) titers, and the responses of T follicular helper (TFH) cells and germinal center B (GCB) cells, while also enhancing the capacity of HA-specific CD4^+^ and CD8^+^ T cells to secrete IFN-γ and TNF-α. Following lethal homologous virus challenge, the GM-CSF-Flt3L group exhibited markedly reduced lung viral loads and no significant pathological damage in lung tissues. Conclusions: These findings demonstrate that the GM-CSF-Flt3L fusion adjuvant complementarily enhances humoral and cellular immune responses induced by the influenza DNA vaccine and improves protective efficacy, highlighting its potential as an effective DNA vaccine adjuvant.

## 1. Introduction

DNA vaccines are a novel vaccine technology first conceptualized in the 1990s. Previous studies have reported that naked DNA could express protein in mouse muscle cells and induce specific immune responses without specialized delivery systems [1]. Subsequent studies confirmed that, upon injection into a host, plasmid DNA encoding an antigen utilizes the host’s protein synthesis machinery to produce the antigen while activating the MHC class I pathway, thereby preferentially inducing CD8^+^ T cell-mediated cellular immunity [2]. This approach preferentially primes CD8^+^ T cell responses, distinguishing it from many protein-based vaccines. Indeed, DNA vaccines have shown robust protective efficacy in animal models, including mice, dogs, salmon, and poultry [3]. DNA vaccines offer promising potential in preclinical and clinical studies against viral infectious diseases, such as HIV-1, Ebola, and Zika, and cancers, including prostate, breast, and cervical cancer, owing to their simple manufacturing process and high stability [4,5]. However, few DNA vaccines have successfully advanced to clinical trials, mainly due to poor cellular uptake and low immunogenicity [6]. Thus, further development and optimization of DNA vaccines are urgently needed to overcome these challenges.

Adjuvants, which are components used in combination with vaccine antigens, have core functions that include enhancing antigen immunogenicity, prolonging immune protection, reducing antigen dosage, and potentially modulating the immune response. Adjuvants enhance antigen uptake, processing, and presentation by upregulating the expression of costimulatory molecules and MHC molecules on the surfaces of DCs, thereby providing essential signals for T-cell activation. Thus, DCs serve as the central hub through which adjuvants exert their effects, a role that is particularly critical for DNA vaccines that depend on the MHC class I presentation pathway. Currently, DNA vaccine adjuvants include cytokines, chemokines, and co-stimulatory molecules, notably cytokines that target DCs differentiation and maturation, such as Granulocyte-macrophage colony-stimulating factor (GM-CSF) and Fms-related tyrosine kinase 3 ligand (Flt3L). GM-CSF is a cytokine produced by lymphocytes, macrophages, fibroblasts, and other cells in response to pro-inflammatory cytokines. GM-CSF is capable of binding to its cognate receptor, inducing receptor dimerization and subsequent transphosphorylation of intracellular tyrosine residues, thereby activating downstream signaling pathways and regulating immune responses [7]. GM-CSF has been shown to promote DC maturation and enhance antibody titers in various vaccine studies, including those against HIV, pseudorabies, and Ebola virus [8]. Specifically, GM-CSF increases MHC II expression on CD11b^+^ DCs in the mouse spleen and augments the number of DCs at the injection site [9]. However, GM-CSF exerts a synergistic effect with other cytokines such as IL-6 in exacerbating lung injury under excessive inflammatory conditions elicited by viral infections including COVID-19, thereby driving a cytokine storm and consequent severe organ dysfunction [10]. Therefore, the optimal utilization of GM-CSF in vaccines requires a careful balance between its immunostimulatory capacity and potential pro-inflammatory effects. Flt3L is a cytokine that regulates the proliferation of early hematopoietic progenitor cells and is primarily responsible for maintaining the number and functional status of DCs in peripheral lymphoid organs. The number of DCs in the mouse spleen, lymph nodes, peripheral blood, and lung tissue was markedly elevated following continuous administration of Flt3L recombinant protein [11]. Flt3L promotes BMDC maturation and increases the serum titers of specific IgG antibodies and IFN-γ, thereby reducing post-infection mortality and histopathological damage [12].

Cytokine adjuvants are usually administered as combinations of two cytokines with complementary or synergistic effects, such as the combination of IL-7 with IL-2 or IL-15, which enhances T-cell proliferation [13]. The combined use of GM-CSF and Flt3L produces additive effects, primarily through the coordinated regulation of DC subsets and functions. Co-administration of two plasmids encoding GM-CSF and Flt3L induces the generation of more CD11blow and CD11bhigh DC subsets, and these DCs exhibit significantly higher antigen capture efficiency than those induced by either cytokine alone [14]. In addition, the co-administration of plasmids encoding GM-CSF, Flt3L, and hepatitis C virus (HCV) NS5 enhances antibody responses against the NS5 protein and CD4^+^ T cell proliferation in the HCV DNA vaccine [15]. This combination also promotes enhanced activity of cytotoxic T lymphocytes (CTLs), inhibiting tumor formation and progression in the tumor vaccine [16]. However, co-delivery of a multi-plasmid leads to variable co-transfection efficiencies across cells. Some cells may incorporate only a subset of the plasmids, giving rise to disparate expression levels of the two proteins. In contrast, a fusion gene ensures that multiple proteins are expressed simultaneously at equimolar ratios within the same cell, overcoming expression limitations caused by differences in transfection efficiency in multi-plasmid systems. Furthermore, compared with IRES-based bicistronic systems, the fusion protein avoids the expression imbalance in which downstream gene expression is only 20–50% of that of the upstream gene [17]. This design preserves the biological activity of the fusion protein. For example, the mRNA-encoded fusion cytokine GIFT4, which consists of GM-CSF and IL-4, protects mice against influenza A virus infection [18]. Although the additive effect of GM-CSF and Flt3L as adjuvants has been demonstrated in various vaccines, whether this additive effect is preserved or enhanced by a chimeric GM-CSF-Flt3L fusion design in an influenza DNA vaccine remains unclear [18].

In this study, we constructed a GM-CSF-Flt3L expression plasmid encoding a fusion protein of GM-CSF and Flt3L to evaluate whether this adjuvant retains the immunomodulatory functions of each individual cytokine. BALB/c mice were immunized with the GM-CSF, Flt3L, or GM-CSF-Flt3L adjuvant plasmid together with the influenza virus HA-Foldon antigen plasmid via intramuscular injection combined with electroporation. We then evaluated the effects of the different adjuvants on DC activation and recruitment, as well as on humoral and cellular immune responses. Finally, we validated the protective efficacy of these adjuvants in a homologous influenza virus challenge model.

## 2. Materials and Methods

### 2.1. Cell and Virus Culture

The MDCK cell line was kindly provided by Professor Lu Lu (School of Basic Medical Sciences, Fudan University), and 293T cells were purchased from the American Type Culture Collection (ATCC, CRL-3216). Both cell lines were cultured in DMEM complete medium, which was prepared using DMEM (Meilunbio, Dalian, China, MA0212) supplemented with 10% fetal bovine serum (FBS, vivacell, Shanghai, China, C04001) and 1% penicillin streptomycin (Gibco, Waltham, MA, USA, 15140122). Cells were maintained at 37 °C in a humidified atmosphere with 5% CO_2_.

Influenza A virus (A/Puerto Rico/8/1934 (H1N1)) was kindly provided by Professor Lu Lu. The virus was propagated in the allantoic cavity of 9-day-old specific pathogen-free (SPF) chicken embryos (Boehringer, Beijing, China) at 35 °C. After 72 h, the allantoic fluid was collected by centrifugation at 6000 r/min for 10 min at 4 °C to remove debris, aliquoted, and stored at −80 °C.

### 2.2. Animals

Female BALB/c mice (6–8 weeks old) were obtained from Beijing Vital River Laboratory Animal Technology Co., Ltd. (Beijing, China). STOCK Tg(CAG-KikGR)33Hadj/J transgenic mice (hereafter referred to as KikGR mice) were purchased from The Jackson Laboratory and bred in-house to 6–8 weeks of age. Mice were housed in a temperature-and humidity-controlled environment with free access to food and water. All animal procedures were approved by the Institutional Animal Care and Use Committee of the School of Basic Medical Sciences, Fudan University (Ethics approval number: 20230301-39).

### 2.3. Recombinant Plasmid Construction

The GM-CSF recombinant plasmid was constructed using the mouse GM-CSF gene sequence (GenBank accession No. NP_034099) as a template, and the Flt3L recombinant plasmid was constructed based on the Flt3L gene sequence (GenBank accession No. ACZ54110.1). For the GM-CSF-Flt3L recombinant plasmid, the C-terminus of GM-CSF was linked to the Flt3L gene via a flexible (GGGS)_3_ linker, and a Fibritin trimerization motif [19] was introduced between them to promote trimer formation. A secretion signal peptide [20] was added to the N-terminus of each gene, and codons were optimized for mammalian cell expression. A Kozak consensus sequence was added before the start codon. The target genes were cloned into the pVAX1 eukaryotic expression vector and confirmed by Sanger sequencing. All gene fragments were synthesized by GenScript Biotech Corporation (Nanjing, China). The construction of the HA-Foldon plasmid has been described previously [20].

### 2.4. Cell Transfection

Recombinant plasmids were transformed into DH5α competent cells(WEIDI, DL1001), and endotoxin-free plasmid DNA was extracted using an endotoxin-free maxi prep kit (Magen, Guangzhou, China, P1156-02). One day before transfection, 293T cells were seeded into 10 cm dishes. When cells reached approximately 80% confluency, transfection was performed. The diluted plasmid was mixed with a transfection reagent (Shanghai Life-iLab Biotech Co., Ltd., Shanghai, China, AC04L092), incubated at room temperature for 30 min, and then added dropwise to the 293T cells. At 48 h post-transfection, the cell supernatant and cells were separated by centrifugation at 10,000 r/min for 30 min at 4 °C. The cell pellet was lysed for RNA extraction. The cell supernatant was concentrated using 4 kDa ultrafiltration tubes (Millipore, Billerica, MA, USA, UFC903096) at 4 °C and 2000 rpm for 10 min.

### 2.5. Liquid Chromatography Tandem Mass Spectrometry (LC-MS/MS)

LC-MS/MS experiments were performed using an Orbitrap Exploris 480 mass spectrometer coupled with an Easy-nLC 1200 liquid chromatography system (Thermo Fisher Scientific, Waltham, MA, USA). Peptides were separated on a 40 cm C18 reversed-phase column (75 μm inner diameter, Kyoto Monotech, Kyoto, Japan) using a gradient elution. The mass spectrometer was operated in a cycle time mode of 2 s, with a scan range of *m*/*z* 350–1600 and a resolution of 60,000 (at *m*/*z* 200). Precursor ions were fragmented by higher energy collisional dissociation (HCD) with a normalized collision energy (NCE) of 29%, and MS/MS spectra were acquired at a resolution of 15,000 (at *m*/*z* 200). Data were processed using PEAKS Online X, searching against the human SwissProt and mouse INSR databases. Trypsin was specified as the enzyme (allowing up to 3 missed cleavages), with precursor and fragment ion mass tolerances of 10 ppm and 0.02 Da, respectively.

### 2.6. BMDC Isolation and Culture

Bilateral femurs were aseptically removed from 6–8-week-old male C57BL/6 mice, and the bone marrow cavities were flushed with pre-chilled PBS. The flush solution was centrifuged at 1200 r/min for 5 min. After discarding the supernatant, red blood cell lysis buffer was added, and the mixture was incubated for 5 min, followed by another centrifugation to remove the lysis buffer. Cells were then resuspended in complete medium containing 20 ng/mL mouse GM-CSF (Peprotech, Cranbury, NJ, USA, 315-03) and 10 ng/mL mouse IL-4 (Peprotech, Cranbury, NJ, USA, 214-14) and seeded at a density of 1 × 10^6^ cells/mL in cell culture dishes. On day 3 of culture, half of the medium was replaced with fresh complete medium containing the same cytokines. On day 6, cells were collected, resuspended, and adjusted to 1 × 10^6^ cells/mL for replating. Transfected cell supernatants were then added, with Lipopolysaccharide (LPS, InvivoGen, Toulouse, France, TLRL-B5LPS) as a positive control, and the cells were cultured for an additional 48 h before flow cytometric analysis.

### 2.7. Animal Immunization

Female BALB/c mice (6–8 weeks old) received two immunizations (3 weeks apart) on days 0 and 21. A 50 μL volume of SSC buffer (Solarbio, Beijing, China, S1030) containing the respective plasmids was injected intramuscularly into the hind leg, followed immediately by electroporation at the injection site using an electroporator (Inovio, Plymouth Meeting, PA, USA, program OpBlock 0079). The experimental groups were as follows: 33.3 μg pVAX1 (negative control); 25 μg HA-Foldon + 8.3 μg pVAX1 (antigen only); 25 μg HA-Foldon + 8.3 μg Flt3L; 25 μg HA-Foldon + 8.3 μg GM-CSF; and 25 μg HA-Foldon + 8.3 μg GM-CSF-Flt3L. For each time point, 4-6 mice were used per group. Depending on the assay, samples were collected at different time points: draining lymph nodes on day 3 after the first immunization for innate immune analysis; blood at 2 weeks after the first and second immunizations for serum isolation and antibody analysis by ELISA and HI assay; draining lymph nodes and spleens at 1 week after the second immunization for GCB and TFH analysis; spleens at 2 weeks after the second immunization for T-cell immune analysis, and liver, kidney, and heart tissues for safety assessment. Additionally, at 2 weeks after the second immunization, the mice were challenged intranasally with 20 μL containing 10 LD_50_ of A/Puerto Rico/8/1934 (H1N1) virus. On day 5 post-challenge, lung tissues were collected for viral load determination and histopathological analysis, and body weight changes were monitored for 14 days. Mice that lost ≥20% of their initial body weight were euthanized.

The method for detecting migDC using KikGR mice has been described previously [21]. Briefly, female KikGR mice (6–8 weeks old) were depilated on the hind leg, and a 436 nm violet light (200 mW/cm^2^) was applied for 4 min to induce photoconversion. Mice were then immunized using the same immunization protocol, and draining lymph nodes were collected on day 2 after the first immunization for subsequent analysis.

### 2.8. Flow Cytometry

Immediately after euthanasia, draining lymph nodes and spleens were collected and processed into single-cell suspensions by mechanical grinding and red blood cell lysis. For surface marker staining, single-cell suspensions were incubated with a cocktail of fluorochrome-conjugated antibodies (antibody clones, fluorochromes, and gating strategies are detailed in Supplementary Materials) at room temperature for 15 min. After incubation, cells were centrifuged at 800× *g* for 3 min at 4 °C, the supernatant was discarded to remove unbound antibodies, and the cells were resuspended in 200 µL of pre-chilled FACS buffer.

For analysis of IFN-γ and TNF-α production by T cells, 1 × 10^6^ splenocytes per well were seeded and stimulated with HA-specific T-cell peptides (FERFEIFPK, SVSSFERFEIFPK) at 37 °C in 5% CO_2_ for 10 h in the presence of 1:1000 diluted Golgi transport inhibitor (BD Biosciences, San Jose, CA, USA, 554724). Cells were then stained for surface markers, fixed and permeabilized using Foxp3/transcription factor staining buffer set for 15 min, and finally incubated with an intracellular cytokine antibody cocktail at room temperature for 1 h. After centrifugation, cells were immediately filtered through a 70 µm cell strainer into flow cytometry tubes and analyzed on a flow cytometer (BD Biosciences, San Jose, CA, USA, LSRFortessa).

### 2.9. ELISA

ELISA was performed using 96-well plates (Corning, Corning, NY, USA, 3590). Plates were coated with 2 μg/mL HA protein without the Foldon tag overnight at 4 °C, washed with PBST, and blocked with 5% non-fat milk for 1 h. Mouse sera were serially diluted starting from 1:100, added to the plates, and incubated at 37 °C for 1.5 h. HRP-conjugated anti-mouse IgG (Thermo, Waltham, MA, USA, C31430100), IgG2a (Southern Biotech, Birmingham, AL, USA, 1155-05), or IgG1 (Southern Biotech, Birmingham, AL, USA, 1144-05) secondary antibodies were diluted 1:5000 and incubated at 37 °C for 1 h. After washing with PBST, TMB substrate (Tiangen, Beijing, China, PA107-02) was added for 15 min for color development, and the reaction was stopped with 1 M sulfuric acid. Absorbance was read at 450 nm with a reference wavelength of 620 nm using a microplate reader (Bio RAD, Hercules, CA, USA, xMark). Specifically, the cutoff value for ELISA was defined as 2.1 times the mean OD_450_–_620_ of the negative control sera. Samples with OD values equal to or above this cutoff were considered positive, while those below were considered negative. All endpoint titers were log_10_-transformed for data presentation and statistical analysis. For samples with OD values below the cutoff at the starting dilution (1:100), a log_10_-transformed titer of 1.7 was assigned to facilitate visualization and statistical comparisons.

### 2.10. HI Assay

Mouse sera were mixed with 3 volumes of receptor-destroying enzyme (RDE, Seiken, Tokyo, Japan, 340122) and incubated at 37 °C for 18 h, followed by inactivation at 56 °C for 30 min. Six volumes of normal saline and 0.5 volumes of guinea pig red blood cells (HongQuan Bio, Guangzhou, China, HQ80077-001) were then added, and the mixture was shaken at 4 °C for 1 h. After centrifugation, the supernatant was collected. Treated sera were serially diluted 2-fold in PBS in 96-well U-bottom plates, mixed with an equal volume of 4 hemagglutination units of virus, and incubated at 25 °C for 30 min. An equal volume of 1% guinea pig red blood cells was added and incubated at 25 °C for 40 min. The HI titer was defined as the highest dilution of serum that completely inhibited hemagglutination; that is, the formation of a button of red blood cells at the bottom of the well.

### 2.11. Quantitative PCR

Total RNA was extracted from transfected 293T cells using an RNA extraction kit (SparkJade, Jinan, China, AC0202-A). Viral RNA from lung tissue homogenates was extracted using a viral RNA extraction kit (CWBio, Taizhou, China, CW0586S). Extracted RNA samples were reverse transcribed into cDNA with one-step RT-gDNA digestion SuperMix (Yeasen, Shanghai, China, 11150ES10). qPCR was performed using the following specific primers: Flt3L (forward: 5′-GGATCGAGCAGCTGAAGACC-3′, reverse: 5′-GCTGGAATGTGCAGCTTGTC-3′), GM-CSF (forward: 5′-GCCCTGAACCTGCTTGATGA-3′, reverse: 5′-AGGAGAACTCGTTGCTCACC-3′), GAPDH (forward: 5′-CTTTGGTATCGTGGAAGGACTC-3′, reverse: 5′-GTAGAGGCAGGGATGATGTTC-3′), and influenza virus M2 (forward: 5′-GACCAATCCTGTCACCTCTGAC-3′, reverse: 5′-AGGGCATTTTGGACAAAGCGTCTA-3′). The qPCR reaction was run on (Bio RAD, CFX 96 Touch real-time PCR) under the following conditions: initial denaturation at 95 °C for 30 s, followed by 40 cycles of 95 °C for 10 s and 60 °C for 30 s. Each sample was run in triplicate technical replicates, and the mean value was used for analysis. The 2^−ΔΔCt^ method was used for relative quantification of Flt3L and GM-CSF transcripts. For M2, a standard curve was generated using a plasmid standard containing different copy numbers; the standard curve was constructed with the logarithm of the copy number of the standard as the x-axis and the measured Ct value as the y-axis.

### 2.12. H&E Staining

Lung, kidney, liver, and heart tissues were fixed in 4% paraformaldehyde for 48 h, then embedded in paraffin, sectioned, and stained with hematoxylin and eosin (H&E). Images were acquired using a fluorescence inverted microscope (Advanced Microscopy Group, Bothell, WA, USA, EVOS AMG). The severity of lung lesions was graded from I to V based on the extent of inflammatory cell infiltration: I, no significant pathological changes; II, perivascular or parenchymal infiltration involving less than 25% of the lung lobe area; III, infiltration involving 25–50% of the lung lobe area; IV, infiltration involving 50–75% of the lung lobe area; and V, infiltration involving more than 75% of the lung lobe area.

### 2.13. Statistical Analysis

All data are presented as mean ± standard deviation (SD). Comparisons between groups were performed using one-way ANOVA or two-way ANOVA. When the overall test was statistically significant, Tukey’s post-hoc test was used for multiple comparisons. A *p*-value < 0.05 was considered statistically significant. All statistical analyses and graph generation were performed using GraphPad Prism software (version 9.5.0). Some figures created in Biorender. H.L. (2026) https://BioRender.com were optimized using BioRender (www.biorender.com).

## 3. Results

### 3.1. Design and Construction of the GM-CSF-Flt3L Expression Plasmids

**Figure 1:**
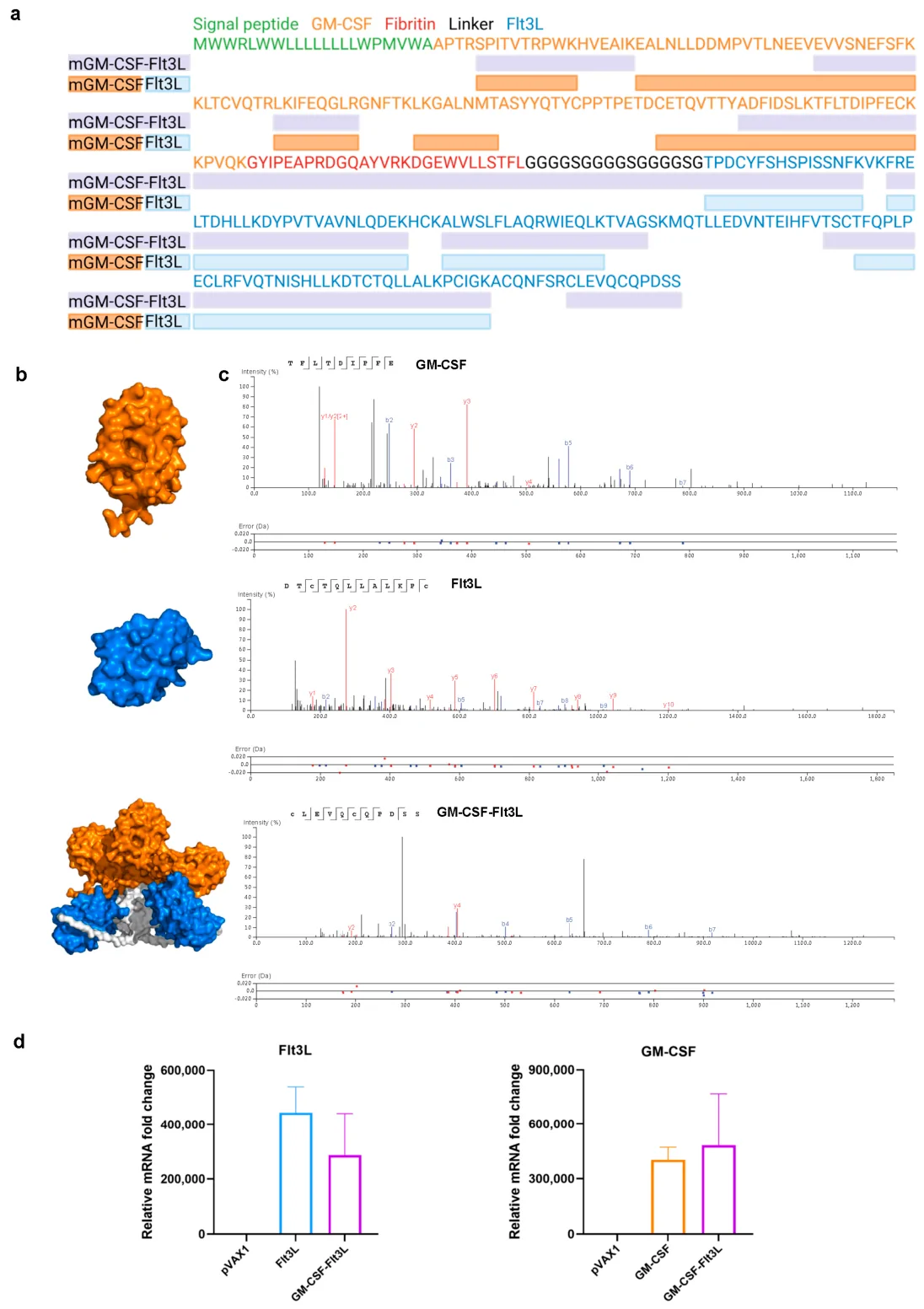
Design and characterization of GM-CSF, Flt3L and GM-CSF-Flt3L adjuvant. (**a**) Schematic representation of the amino acid sequences of GM-CSF, Flt3L, and the GM-CSF-Flt3L fusion. The upper panel shows the amino acid sequences of GM-CSF alone and Flt3L alone, each using their respective native sequences, as well as the GM-CSF-Flt3L fusion construct, in which the C-terminus of GM-CSF is linked to a (GGGS)_3_ flexible linker, followed by a Fibritin trimerization motif and the Flt3L sequence. Peptide coverage was validated by LC-MS/MS using the supernatant of transfected cells. Peptide fragments are displayed as color-coded bars beneath the amino acid sequences. Orange, blue, and purple represent GM-CSF-derived, Flt3L-derived, and GM-CSF-Flt3L peptides, respectively. (**b**) Predicted structures of GM-CSF (top), Flt3L (middle), and the GM-CSF-Flt3L (bottom) fusion protein by AlphaFold2. (**c**) Identified peptides of GM-CSF (top), Flt3L (middle), and the GM-CSF-Flt3L fusion protein (bottom) by LC-MS/MS analysis. (**d**) qPCR analysis of the Flt3L (left) and GM-CSF (right) genes.

We constructed separate plasmids expressing GM-CSF and Flt3L, respectively, using their respective gene sequences. Additionally, a recombinant plasmid (GM-CSF-Flt3L) was constructed by fusing the C-terminus of GM-CSF to a (GGGS)_3_ flexible linker, followed by a fibritin trimerization motif and the Flt3L sequence. The amino acid sequences of the constructed plasmids are shown in Figure 1a. To validate expression of the proteins from these constructs, each plasmid was transfected into 293T cells, and the cell culture supernatant was analyzed by LC-MS/MS. Peptide mapping analysis confirmed that the detected peptides spanned nearly the entire length of the sequences encoded by the three plasmids, with 100% sequence identity to the expected GM-CSF, Fibritin and Flt3L domains (Figure 1a). Representative MS/MS spectra are shown in Figure 1c. Furthermore, the GM-CSF-Flt3L protein adopts conformations similar to those of native GM-CSF and Flt3L, as predicted by AlphaFold2, suggesting enhanced structural stability (Figure 1b). In transfected cells, no GM-CSF or Flt3L transcripts were detected in the pVAX1 group. Flt3L transcripts were detected in the Flt3L and GM-CSF-Flt3L groups, whereas GM-CSF transcripts were detected in the GM-CSF and GM-CSF-Flt3L groups (Figure 1d). These results demonstrated that all the plasmids were transcribed and translated in mammalian cells.

### 3.2. GM-CSF-Flt3L Adjuvant Plasmid Recruits and Activates DCs

**Figure 2:**
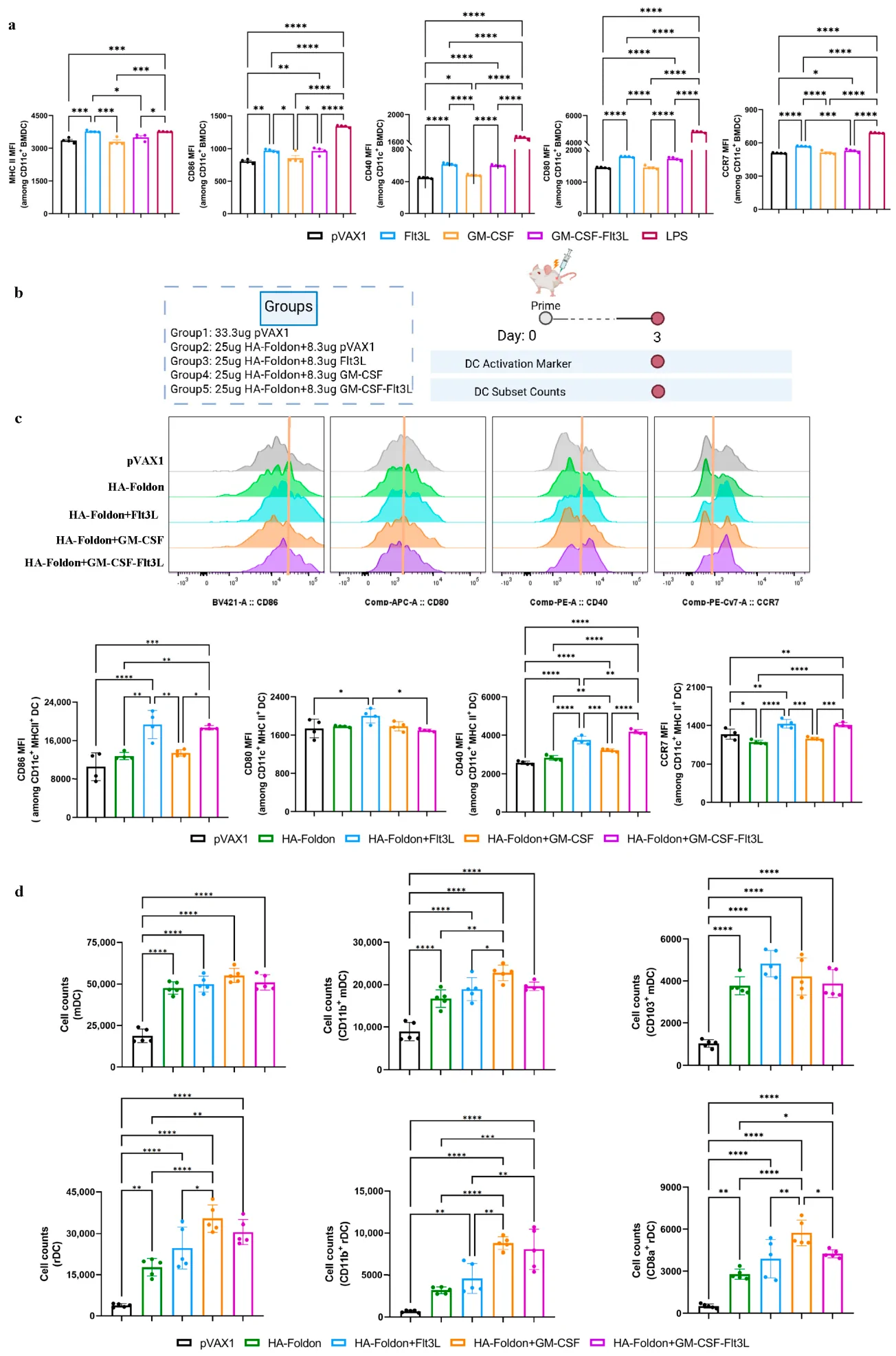
DCs are recruited and activated by the GM-CSF-Flt3L adjuvant plasmid. (**a**) The MFI values analysis of CD80, CD86, CD40, CCR7, and MHC II on CD11c^+^ BMDCs by flow cytometry. The gating strategy is shown in Figure S2. (**b**) Immunization schedule. BALB/c mice received intramuscular injections of DNA vaccines followed by electroporation at the injection site. The groups were as follows: 33.3 μg pVAX1 (negative control), 25 μg HA-Foldon + 8.3 μg pVAX1 (antigen only), 25 μg HA-Foldon + 8.3 μg Flt3L, 25 μg HA-Foldon + 8.3 μg GM-CSF and 25 μg HA-Foldon + 8.3 μg GM-CSF-Flt3L. Draining lymph nodes were harvested on day 3 post-immunization for analysis of the activation status of MHC II^+^ CD11c^+^ DC and the distribution of DCs subsets by flow cytometry. (**c**) The MFI values analysis of CD86, CD80, CD40, and CCR7 on MHC II^+^ CD11c^+^ DCs by flow cytometry. *n* = 4 mice per group. The gating strategy is shown in Figure S3a. Representative flow cytometry MFI (top) and the corresponding statistical results (bottom) are shown. (**d**) Absolute numbers of migratory DCs (migDC), CD11b^+^ migratory DCs (CD11b^+^ mDC), CD103^+^ migratory DCs (CD103^+^ mDC), resident DCs (resDC), CD11b^+^ resident DCs (CD11b^+^ rDC), and CD8a^+^ resident DCs (CD8a^+^ rDC) by flow cytometry. *n* = 5 mice per group. The gating strategy is shown in Figure S3b. Data points represent individual mice. Results are expressed as mean ± standard error of the mean (SEM). Statistical significance was evaluated using one-way analysis of variance (ANOVA), with significance levels denoted as: ns, not significant; * *p* < 0.05; ** *p* < 0.01; *** *p* < 0.001; **** *p* < 0.0001.

To evaluate the ability of the three adjuvants to activate DCs, mouse BMDCs were isolated and co-incubated for 48 h with supernatants from 293T cells transfected with the respective adjuvant plasmids, followed by flow cytometric detection of activation markers on the surfaces of CD11c^+^ BMDCs. The Flt3L group showed higher expression of MHC II, CD80, CD86, CD40, and CCR7 than the negative control, GM-CSF, and GM-CSF-Flt3L groups. Moreover, MHC II expression in the Flt3L group reached levels comparable to those in the LPS positive control. The GM-CSF-Flt3L group showed higher expression levels of CD80, CD86, and CD40 than the GM-CSF group, but lower than the Flt3L group (Figure 2a).

To further validate the DC-activating effect of the adjuvants in vivo, BALB/c mice were immunized intramuscularly with a mixture of the influenza antigen-encoding DNA vaccine and the respective adjuvant plasmids, followed by electroporation at the injection site (Figure 2b). Draining lymph node cells were collected on day 3 of immunization, and activation markers on MHC II^+^ CD11c^+^ DCs were analyzed by flow cytometry. Increased expression of CD86, CD40, and CCR7 on lymph node DCs was observed in the Flt3L and GM-CSF-Flt3L groups compared with the negative control, antigen-only, and GM-CSF groups, consistent with the BMDCs in vitro. Notably, higher CD40 expression was detected in the GM-CSF-Flt3L group than in the Flt3L group (Figure 2c). Taken together, GM-CSF-Flt3L induced a balanced activation profile, outperforming GM-CSF alone and showing superior CD40 upregulation compared to Flt3L. GM-CSF preferentially expanded resident DC subsets, while Flt3L favored migratory populations, and the fusion provided balanced expansion.

Furthermore, the absolute numbers of different DC subsets were analyzed by flow cytometry. An increased absolute number of resident DCs (resDC), particularly the CD11b^+^ and CD8^+^ subsets, was observed in the GM-CSF group, whereas the expansion effect of GM-CSF-Flt3L on these subsets was lower than that of GM-CSF. Conversely, the expansion of CD103^+^ migratory DCs (migDC) was primarily promoted by the Flt3L group (Figure 2d). The GM-CSF-Flt3L group exhibited an expansion capacity for each DC subset that was intermediate between that of GM-CSF and that of Flt3L, whereas GM-CSF and Flt3L each displayed distinct selective expansion of the DC subsets.

### 3.3. GM-CSF-Flt3L Adjuvant Plasmid Enhances the Recruitment of migDC

**Figure 3:**
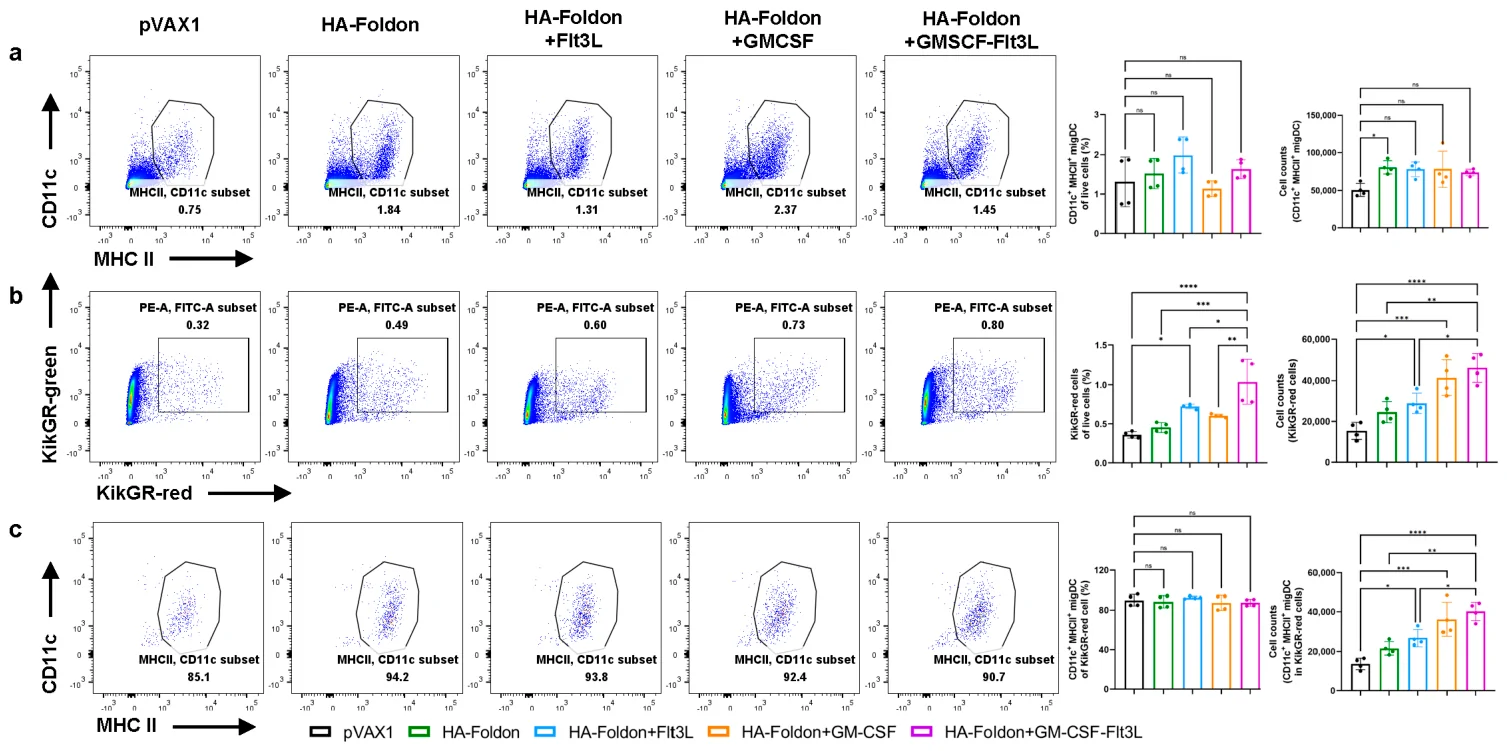
The GM-CSF-Flt3L adjuvant plasmid boosts migDC recruitment. (**a**–**c**) The number and frequency analysis of MHC II^+^ CD11c^+^ migDC (**a**), KikGR-red^+^ cells (**b**), and MHC II^+^ CD11c^+^ cells within the live^+^ KikGR-red^+^ population (**c**) by flow cytometry. *n* = 4 mice per group. The gating strategy is shown in Figure S4. Representative flow cytometry plots (left) and the corresponding statistical results (right) are shown. Data points represent individual mice. Results are expressed as mean ± SEM. Statistical significance was determined by one-way ANOVA, with significance levels denoted as ns, not significant; * *p* < 0.05; ** *p* < 0.01; *** *p* < 0.001; **** *p* < 0.0001.

The photoconvertible KikGR mice were labeled and used to distinguish migDC induced by the adjuvants in vivo [21]. Female KikGR mice of 6–8 weeks of age were depilated on the hind leg muscle, and a 436 nm violet light (200 mW/cm^2^) was applied for 4 min to induce local photoconversion. Then, the mice were immunized with the DNA vaccines via intramuscular injection combined with electroporation, using the same groups and doses as in Figure 2b. Draining lymph nodes were collected at 48 h post-immunization, and the number and percentages of MHC II^+^ CD11c^+^ migDC, photoconverted (KikGR-red^+^) cells, and double-positive cells were analyzed by flow cytometry. Similar results were observed among the groups in terms of both the number and frequency of MHC II^+^ CD11c^+^ migDC. In contrast, both the number and frequency of KikGR-red^+^cells in the GM-CSF-Flt3L group were significantly higher than those in the other groups (Figure 3b). To further validate the cellular identity of the KikGR-red^+^ population, MHC II and CD11c expression was analyzed. Approximately 90% of KikGR-red^+^ cells in each group expressed MHC II^+^ CD11c^+^, indicating that the KikGR-red^+^ population consists mainly of classical migDC (Figure 3c). Moreover, the number of MHC II^+^ CD11c^+^ cells within the KikGR-red^+^ population showed a trend completely consistent with that of the KikGR-red^+^ population, being significantly higher in the GM-CSF-Flt3L group than in the other groups (Figure 3c). The data indicated that GM-CSF-Flt3L significantly enhanced recruitment of photoconverted (KikGR-red^+^) migratory DCs to draining LNs.

### 3.4. GM-CSF-Flt3L Adjuvant Plasmid Boosts Humoral Immune Responses Induced by the HA-Foldon DNA Vaccine

**Figure 4:**
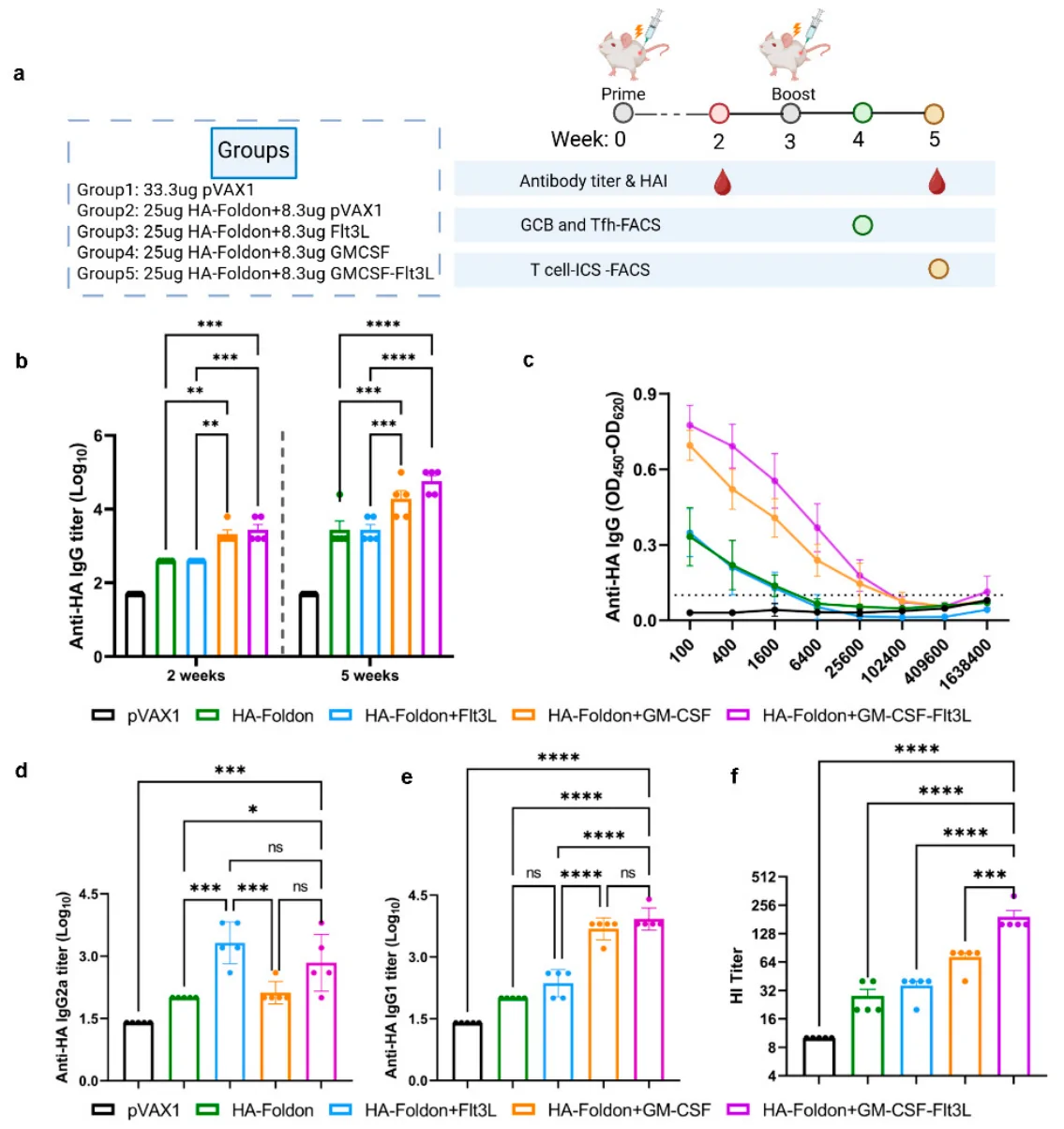
The GM-CSF-Flt3L adjuvant plasmids augment HA-Foldon DNA vaccine-elicited humoral immune responses. (**a**) Immunization schedule. Female BALB/c mice (6–8 weeks old) received two intramuscular electroporation immunizations at a 3-week interval. Sera were collected at 2 weeks after the first and second immunizations. Draining lymph nodes and spleens were collected at 1 week after the second immunization for GCB and TFH analysis. Spleens were also collected at 2 weeks after the second immunization for T cell cytokine secretion analysis. (**b**) HA-specific serum IgG endpoint titers by ELISA. *n* = 5 mice per group. Sera collected at 2 weeks after the first and second immunizations were assessed for endpoint titers. (**c**) Serum dilution curves after the second immunization. The dashed line indicates the cutoff value. (**d**,**e**) HA-specific IgG subclass analysis by ELISA. Serum IgG2a (**d**) and IgG1 (**e**) antibody levels were measured at 2 weeks after the second immunization. (**f**) Homologous virus HI antibody titers. HI titers against the A/Puerto Rico/8/1934 (H1N1) influenza virus strain were measured in sera collected 2 weeks after the second immunization. *n* = 5 mice per group. The dashed line marks the protective threshold at an HI titer of 40. Data points represent individual mice. Results are shown as mean ± SEM. Statistical significance was determined by two-way ANOVA (**b**) or one-way ANOVA (**d**,**f**), with significance thresholds: ns, not significant; * *p* < 0.05; ** *p* < 0.01; *** *p* < 0.001; **** *p* < 0.0001.

To evaluate the immunoenhancing effect of GM-CSF, Flt3L, and GM-CSF-Flt3L as adjuvant plasmids, BALB/c mice were immunized using the same schedule as in Figure 2b, with two vaccinations administered 3 weeks apart (Figure 4a). Sera were collected at 2 weeks after the first and second immunizations, and HA-specific IgG antibody responses were measured by ELISA. The HA IgG endpoint titers in the GM-CSF and GM-CSF-Flt3L groups were not significantly different from each other, and both were significantly higher than those in the Flt3L and antigen-only groups after both immunizations (Figure 4b). Although the GM-CSF and GM-CSF-Flt3L groups showed no statistical difference, the GM-CSF-Flt3L group yielded higher OD_450–620_ values than the GM-CSF group across nearly all dilutions in the serum dilution curves after the second immunization (Figure 4c). Meanwhile, IgG1 and IgG2a antibody titers in sera collected at 2 weeks after the second immunization were measured by ELISA. IgG2a titers in the Flt3L group were similar to those in the GM-CSF-Flt3L group, and both were significantly higher than those in the other groups. (Figure 4d). The GM-CSF group showed no significant difference in IgG1 titers from the GM-CSF-Flt3L group, and both groups exhibited significantly higher IgG1 titers than the remaining groups (Figure 4e). Hemagglutination inhibition (HI) antibody titers against the homologous A/Puerto Rico/8/1934 (H1N1) virus strain were measured in sera collected 2 weeks after the second immunization. The GM-CSF-Flt3L group exhibited a higher HI titer (geometric mean titer, GMT = 242.51) than the other adjuvant groups and the antigen-only group. Comparable results were observed among the GM-CSF, Flt3L, and antigen-only groups, with GMTs of 121.26, 91.90, and 45.95, respectively. All groups induced HI titers ≥40, which is considered protective (Figure 4f). Based on these findings, we concluded that the humoral immune responses of the HA-Foldon DNA vaccine are enhanced by GM-CSF-Flt3L adjuvant plasmids.

### 3.5. GM-CSF-Flt3L Adjuvant Plasmid Amplifies the HA-Foldon Vaccine-Induced GCB Cell and TFH Cell Responses

**Figure 5:**
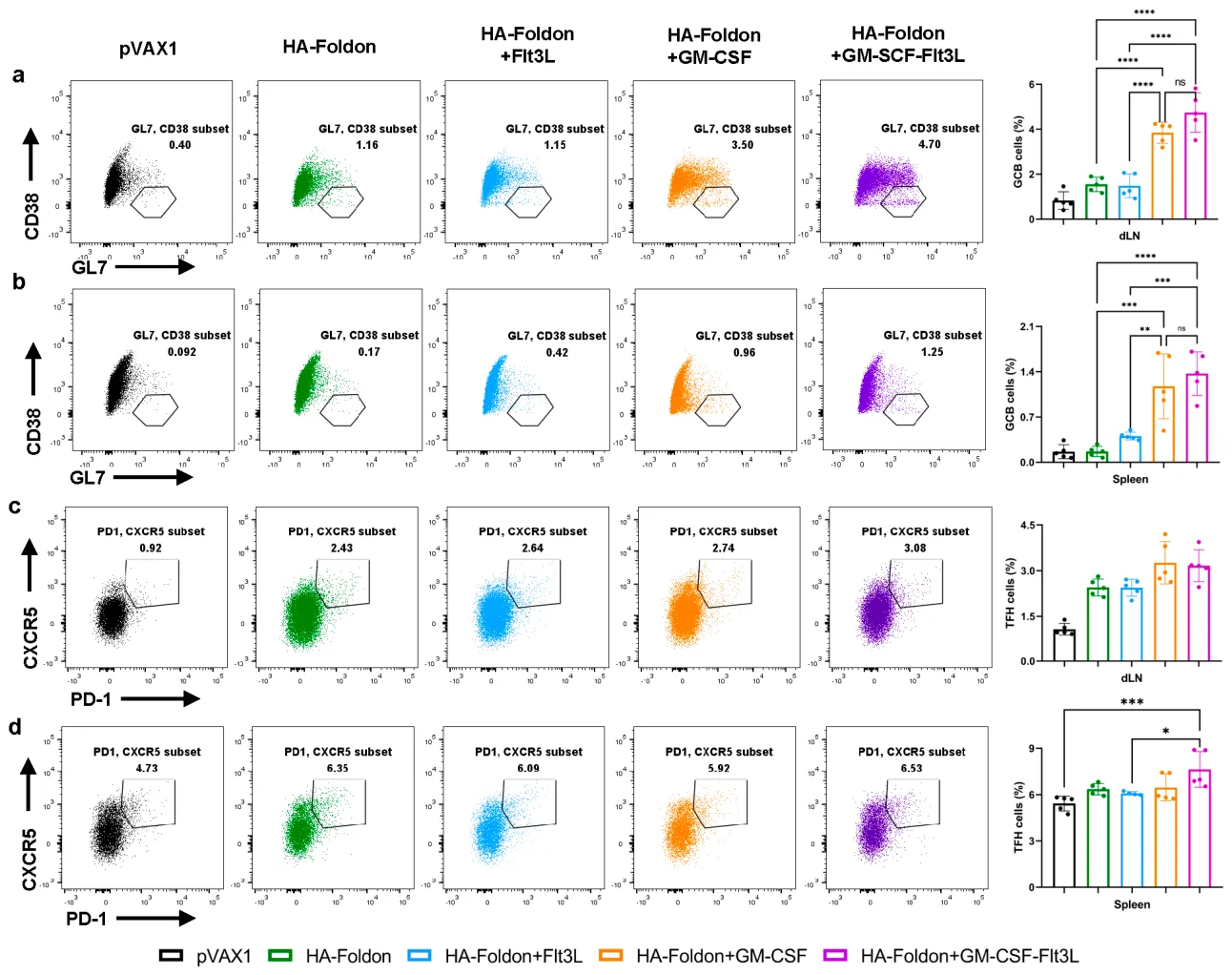
GM-CSF-Flt3L adjuvant plasmid enhances the HA-Foldon vaccine-induced GCB cell and TFH cell responses. (**a**,**b**) The frequencies of GCB cells (B220^+^ GL 7^+^ Fas^+^; gating strategy in Figure S5a) in the draining lymph nodes (**a**) and spleen (**b**) were determined by flow cytometry. (**c**,**d**) The frequencies of TFH cells (CD4^+^ CXCR5^+^ PD 1^+^; gating strategy in Figure S5b) in the draining lymph nodes (**c**) and spleen (**d**) were determined by flow cytometry. Representative flow cytometry plots (left) and the corresponding statistical results (right) are shown. Mice were immunized as outlined in Figure 4a. *n* = 5 mice per group. Data points represent individual mice. Results are expressed as mean ± SEM. Statistical significance was determined by one-way ANOVA, with significance levels denoted as: ns, not significant; * *p* < 0.05; ** *p* < 0.01; *** *p* < 0.001; **** *p* < 0.0001.

GC is a key site for B-cell maturation, and the intensity of the GC response is closely associated with vaccine protective efficacy. TFH cells, as a major regulatory component within the GC microenvironment, directly support the proliferation, differentiation, and isotype switching of GCB cells by providing activation signals such as CD40L and IL-21. To evaluate the regulatory effect of different adjuvants on the GC response, mouse lymph nodes and spleens were collected at 1 week after the second immunization, and the frequencies of GCB cells and TFH cells were analyzed by flow cytometry. A similar pattern was observed for the frequencies of GCB cells in the lymph nodes and spleen. Specifically, the GM-CSF group exhibited higher GCB cell frequencies than the Flt3L, antigen-only, and negative control groups, while showing no significant difference from the GM-CSF-Flt3L group (Figure 5a,b). No significant differences in the frequency of TFH cells in the lymph nodes were detected among the experimental groups (Figure 5c). However, the frequency of TFH cells in the spleen was comparable between the GM-CSF-Flt3L and GM-CSF groups and higher in both groups than in the Flt3L, antigen-only, and negative control groups (Figure 5d). Our results suggested that GM-CSF-Flt3L, similar to GM-CSF, promotes the expansion of TFH cells in the spleen and enhances the GCB response, demonstrating its potential to optimize the GC response.

### 3.6. GM-CSF-Flt3L Adjuvant Plasmid with the HA-Foldon Vaccine Elicits HA-Specific T-Cell Immune Responses

**Figure 6:**
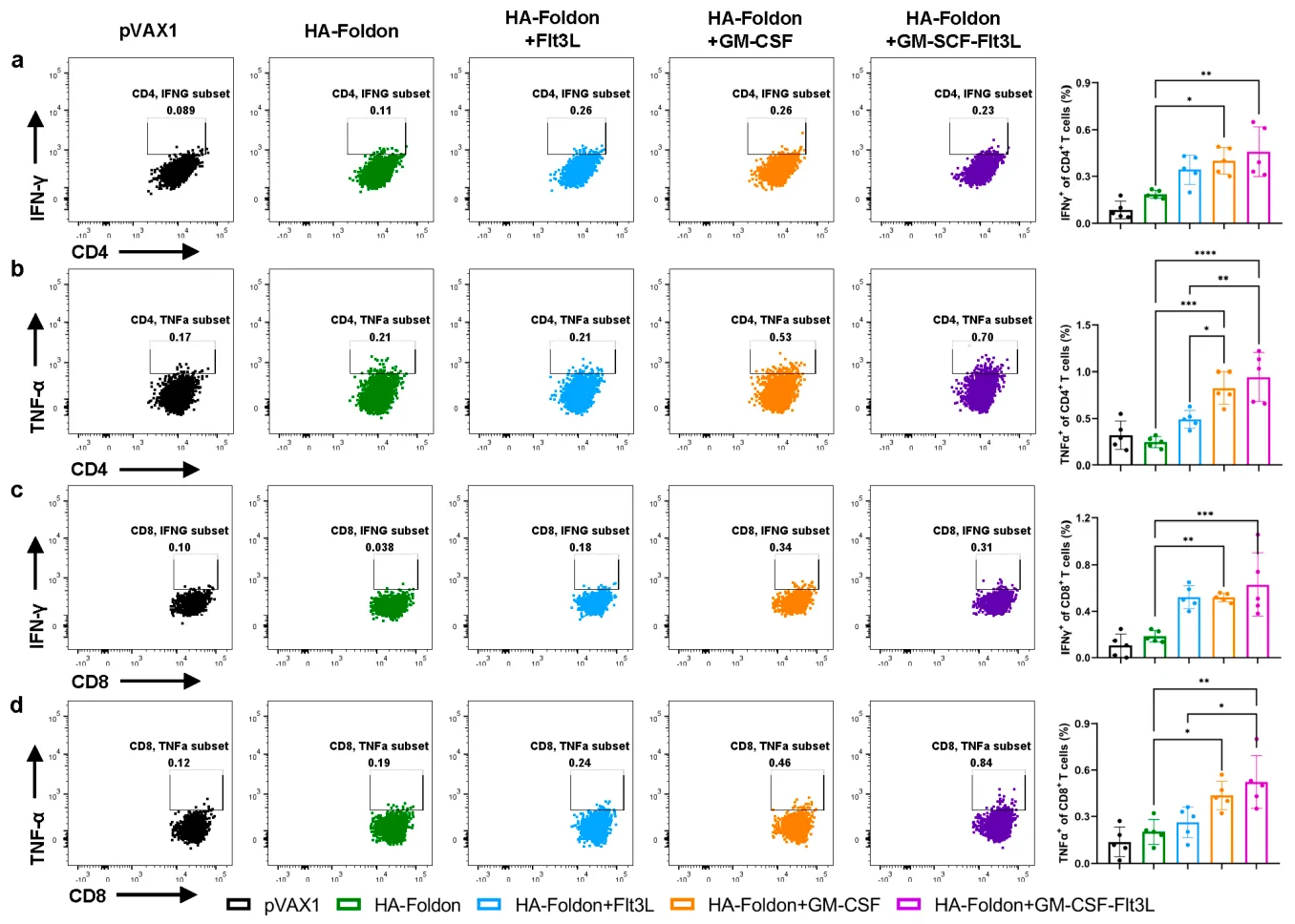
GM-CSF-Flt3L adjuvant plasmid elicits the HA-Foldon vaccine HA-specific T-cell immune responses. (**a**–**d**) The frequency analysis of IFN-γ^+^ CD4^+^ T cells (**a**), TNF-α^+^ CD4^+^ T cells (**b**), IFN-γ^+^ CD8^+^ T cells (**c**), and TNF-α^+^ CD8^+^ T cells (**d**) by flow cytometry. The gating strategy is shown in Figure S6. Representative flow cytometry scatter plots are presented in the left panels, whereas the corresponding quantitative results are displayed in the right panels. Mice were immunized according to the protocol described in Figure 4a. Each experimental group consisted of five mice (*n* = 5). Individual data points represent values from single mice. All results are expressed as mean ± SEM. Statistical comparisons were performed using one-way ANOVA, with significance thresholds defined as: * *p* < 0.05; ** *p* < 0.01; *** *p* < 0.001; **** *p* < 0.0001.

CD4^+^ T cells mediate adaptive immune responses, whereas CD8^+^ T cells exert direct cytotoxic activity against virus-infected cells by secreting effector cytokines, most notably IFN-γ and TNF-α. We collected splenocytes from mice at 2 weeks after the second immunization to determine the effect of different adjuvants on T-cell function induced by vaccination. Splenocytes were stimulated with known HA-specific T-cell epitope peptides of the A/Puerto Rico/8/1934 (H1N1) virus, and the frequencies of IFN-γ^+^ and TNF-α^+^ cells within HA-specific CD4^+^ and CD8^+^ T-cell populations were analyzed by flow cytometry. The GM-CSF-Flt3L group, similar to the Flt3L and GM-CSF groups, induced comparable frequencies of IFN-γ^+^ CD4^+^ and IFN-γ^+^ CD8^+^ T cells, with all groups showing higher frequencies than the antigen-only group (Figure 6a,c). The GM-CSF-Flt3L and GM-CSF groups induced comparable frequencies of TNF-α^+^ CD4^+^ and TNF-α^+^ CD8^+^ T cells, both significantly higher than those in the antigen-only group (Figure 6b,d). Collectively, these results revealed that GM-CSF-Flt3L combines the advantages of both adjuvants, effectively enhancing the effector function of antigen-specific CD4^+^ and CD8^+^ T cells and promoting the production of IFN-γ and TNF-α.

### 3.7. GM-CSF-Flt3L Adjuvant Plasmid Enhances Immune Protection Against Homologous Influenza Virus Challenge Induced by the HA-Foldon Vaccine

**Figure 7:**
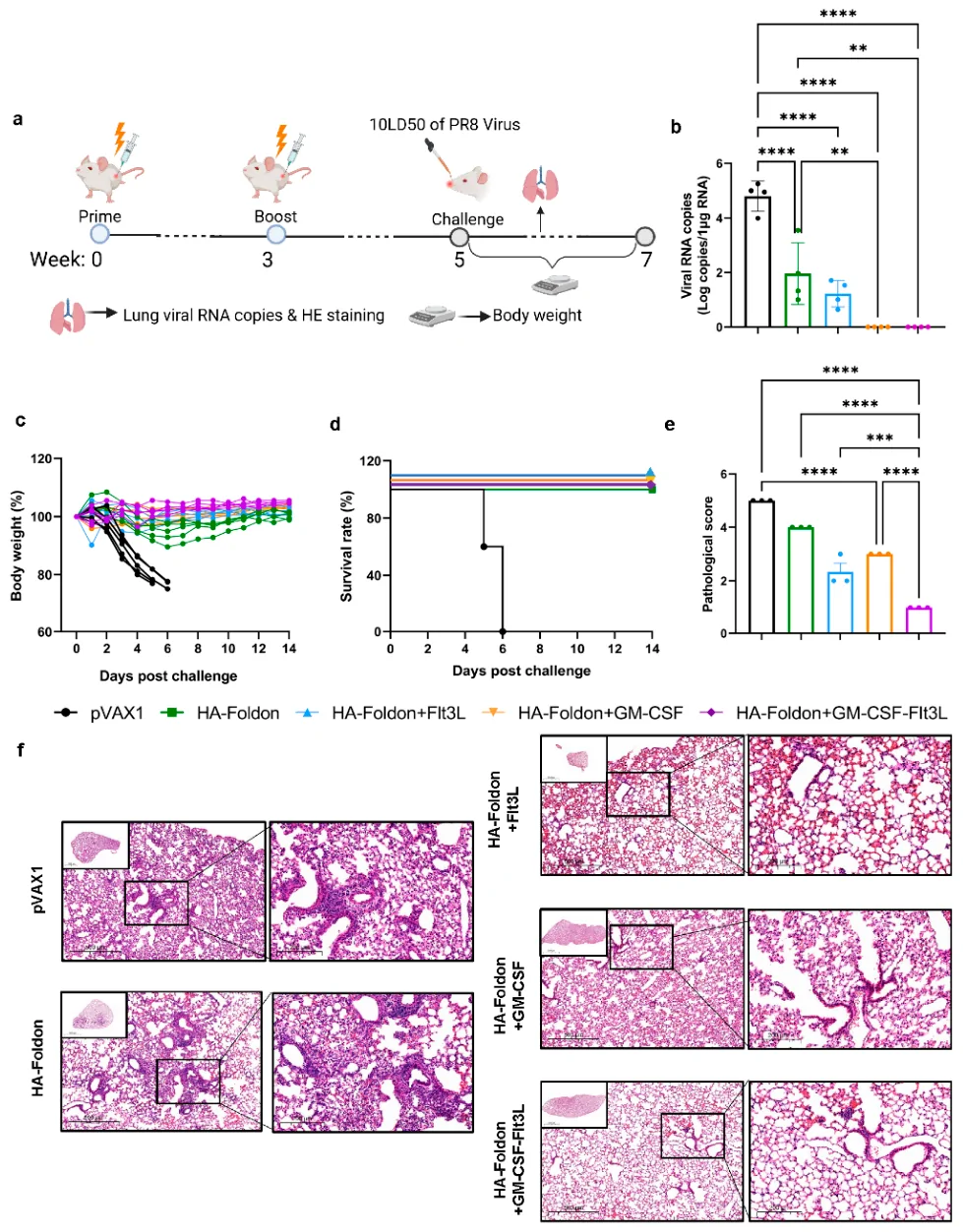
GM-CSF-Flt3L adjuvant plasmid augments the HA-Foldon vaccine-induced immune protection against homologous influenza virus challenge. (**a**) Experimental design for immunization and viral challenge. Female BALB/c mice (6–8 weeks old) received two intramuscular electroporation-based immunizations spaced 3 weeks apart. 2 weeks after the booster dose, mice were intranasally challenged with 20 μL of 10 LD_50_ A/Puerto Rico/8/1934 (H1N1) virus. On day 5 post-challenge, mice were euthanized, and lung tissues were harvested for viral load quantification and histopathological assessment. (**b**) qPCR analysis of pulmonary viral load on day 5 post-challenge (*n* = 5). (**c**,**d**) Post-challenge body weight changes (**c**) and survival profiles (**d**) were monitored from the day of challenge through day 14 post-challenge (*n* = 5). (**e**,**f**) Lung histopathological scores (**e**) and representative H&E staining images (**f**) on day 5 post-challenge were assessed, with histological parameters scored according to the criteria described in Methods for three mice per group. Data points represent individual mice. All results are expressed as mean ± SEM. Statistical comparisons were performed using one-way ANOVA, with significance levels defined as ** *p* < 0.01; *** *p* < 0.001; **** *p* < 0.0001.

We intranasally administered 20 μL of A/Puerto Rico/8/1934 (H1N1) virus containing 10 LD_50_ at 2 weeks post-second immunization in order to evaluate the protective efficacy of different adjuvants against homologous influenza virus challenge (Figure 7a). After euthanizing the mice on day 5 post-challenge, lung tissues were collected for viral load measurement by qPCR. The results showed that the highest viral load was detected in the negative control group, with a mean value of 104.80 copies/μg RNA. The viral load was significantly reduced in the Flt3L group (101.22 copies/μg RNA) but was not significantly different from that in the antigen-only group (101.96 copies/μg RNA). In comparison, the viral loads in both the GM-CSF-Flt3L and GM-CSF groups were not detected (Figure 7b). Body weight changes were monitored daily for 14 days post-challenge, and mice with a weight loss exceeding 20% of their initial body weight were euthanized. The results revealed that only the negative control group displayed continuous weight loss, with all mice dying by day 7 post-challenge. The antigen-only group showed a transient and mild weight loss between days 3 and 6, but all mice survived. No weight loss or mortality was observed in any of the adjuvant groups (Figure 7c,d). Lung tissues collected on day 5 post-challenge were subjected to H&E staining and pathological evaluation. The severity of lung lesions was graded from I to V based on the extent of inflammatory involvement, with higher grades indicating more severe lung damage. The observations showed that the negative control group displayed extensive perivascular and peribronchial inflammatory cell infiltration, accompanied by marked interstitial pneumonia. In the antigen-only group, interstitial inflammation was less severe, but considerable perivascular and peribronchial infiltration persisted. Mild inflammation, restricted to focal areas, was detected in the GM-CSF and Flt3L groups. Notably, the GM-CSF-Flt3L group preserved a largely normal lung tissue architecture, with no apparent pathological changes (Figure 7e,f). In conclusion, these results demonstrated that the combination of GM-CSF and Flt3L exerts a complementary effect, leading to reduced pulmonary viral loads, attenuated lung inflammation, and strengthened immune protection against influenza virus challenge in mice.

To evaluate the safety of the DNA vaccine in vivo, mouse liver, kidney, and heart tissues were collected at 2 weeks after the second immunization for H&E staining and histopathological analysis. These tissue sections showed no inflammatory cell infiltration, hemorrhage, or other obvious histopathological changes in any immunization group (Figure S1), indicating that the adjuvants did not induce toxicity of the DNA vaccines in major organs.

## 4. Discussion

In this study, a GM-CSF-Flt3L fusion adjuvant plasmid complementarily enhanced the immunogenicity and protective efficacy of an HA-Foldon influenza DNA vaccine. Relative to single-adjuvant plasmids, the fusion construct achieved balanced DC activation (superior CD40 upregulation vs. Flt3L; overall better than GM-CSF), effective migratory DC recruitment, robust humoral and GC responses, polyfunctional T-cell activity, and superior lung protection upon homologous challenge.

DCs initiate adaptive immune responses and serve as a bridge connecting innate and adaptive immunity. Flt3L and GM-CSF are two key cytokines that exert distinct regulatory effects on DC differentiation and function. Flt3L primarily acts on hematopoietic progenitor cells through the Flt3 receptor signaling pathway to expand multiple DC subsets of both lymphoid and myeloid origin, including cDC1, cDC2, and pDC. In contrast, GM-CSF acts mainly on myeloid progenitors, preferentially promoting the generation of myeloid-derived DCs, predominantly CD11b^+^ myeloid DCs [22]. We confirmed these regulatory differences by flow cytometry, which showed that the Flt3L group expanded CD103^+^ migDC in draining lymph nodes, whereas the GM-CSF group mainly increased the absolute numbers of resDCs, particularly the CD11b^+^ and CD8^+^ subsets (Figure 2d). CD103^+^ DCs are a cross-presenting DC subset that activates CD8^+^ T cells [23] and have been associated with alleviating inflammatory tissue damage [24]. Accordingly, the Flt3L group showed significantly reduced histopathological lung inflammation after influenza virus challenge (Figure 7f). While we cannot exclude direct contributions from humoral or CD8^+^ T-cell responses, it is plausible that the Flt3L-induced expansion of CD103^+^ DCs contributed to the observed tissue protection. GM-CSF-Flt3L promoted the expansion of multiple DC subsets, combining the advantages of both adjuvants, although it also induced a more complex DC subset distribution pattern compared to single cytokines. Consistent DC activation was observed with GM-CSF-Flt3L in both C57BL/6 mouse BMDCs and BALB/c mouse lymph node DCs (Figure 2a,c). Of note, conventional surface markers may not accurately reflect true migDC [25]. The recruitment of migDC to draining lymph nodes was significantly enhanced by GM-CSF-Flt3L, as demonstrated by flow cytometry using the KikGR mice (Figure 3c). Additionally, some migDC originate from Langerhans cells (LCs) in the skin [26], and LCs can effectively drive TFH and B-cell responses [27], suggesting that GM-CSF-Flt3L-induced migDC may be involved in initiating the GC response.

Humoral immunity is an important indicator of vaccine efficacy, with antibody titers, subclasses, and neutralizing capacity directly determining protective efficacy. We found that GM-CSF and GM-CSF-Flt3L enhanced HA-specific IgG antibody levels and HI titers, with GM-CSF-Flt3L showing a greater enhancement (Figure 4b,f). This finding aligns with the known capacity of GM-CSF to enhance antibody responses in an inactivated Pseudorabies virus (PRV) vaccine [28]. However, GM-CSF exerted no significant effect on antibody responses in an HCV C DNA vaccine and even inhibited antibody responses and protective efficacy in a Japanese encephalitis virus (JEV) prM/E DNA vaccine [29]. These differential effects suggest that the effect of GM-CSF as an adjuvant on humoral immune responses may vary depending on the antigen. In parallel, compared with GM-CSF, Flt3L significantly enhanced HA-specific IgG2a responses (Figure 4d), consistent with the finding that Flt3L induces high levels of specific IgG2a antibodies in a recombinant protein OVA vaccine [30]. Furthermore, Flt3L may drive IgG2a class switching in B cells, a process potentially regulated by cytokines such as IL 12 secreted by lymphoid DC1 subsets expanded by Flt3L [31]. By contrast, GM-CSF may favor a Th2-biased DC polarization possibly involving pathways such as NF-κB/IRF-4, thereby eliciting elevated IgG1 antibody levels [32]. GM-CSF-Flt3L, influenced by both GM-CSF and Flt3L, elicited high levels of both IgG2a and IgG1 antibodies (Figure 4d,e). The underlying details require further validation in subsequent studies.

GC serves as the principal site for the somatic hypermutation and affinity maturation of B cells, with the GC response being tightly orchestrated by TFH cells. Both GM-CSF and GM-CSF-Flt3L outperformed Flt3L in promoting splenic GCB and TFH responses, inducing higher frequencies of these cells at 1 week post-second immunization (Figure 5b,d). This finding echoes a COVID-19 RBD vaccine report showing that GM-CSF elicits robust GC responses [33]. Moreover, the early GCB and TFH responses in the spleen correlated with the later HA-specific IgG antibody levels and HI titers (Figure 4b,f), supporting that the magnitude of the GCB response reflects vaccine-induced humoral immunity, as GCB cells are an early marker of immune responsiveness [34].

T cells directly participate in viral clearance by secreting IFN-γ and TNF-α during influenza virus infection. GM-CSF and GM-CSF-Flt3L significantly enhanced the ability of HA-specific TNF-α^+^ CD4^+^ and TNF-α^+^ CD8^+^ T cells compared with Flt3L, as determined by flow cytometry (Figure 6b,d). We postulate that GM-CSF drives the expansion of myeloid DCs, particularly the CD11b^+^ subset. These DCs orchestrate inflammatory crosstalk between DCs and T cells [35]. This crosstalk, in turn, enables polyfunctional T cells to mount enhanced TNF-α production, which ultimately accelerates viral clearance. Consistent with this, the combination of Flt3L and GM-CSF induces DCs that more effectively activate tumor-specific CD8^+^ T cells and reshape the immune microenvironment [36].

A notable finding of this study is that all vaccinated groups survived the lethal challenge (Figure 7d), yet only the single-adjuvant groups displayed residual lung pathology, while the fusion adjuvant group achieved nearly complete protection from tissue damage (Figure 7e,f). This indicates that, while a single adjuvant can prevent mortality, likely through antibody-mediated viral control [37], the GM-CSF-Flt3L combination is required to suppress local inflammation and preserve lung architecture. From a clinical perspective, reducing disease severity and lung pathology is an important endpoint independent of preventing death [38], and this advantage should be considered when evaluating the translational potential of the fusion adjuvant. Nevertheless, the 10 LD_50_ challenge dose employed in this study may have limited the resolution of protective differences among adjuvanted groups, all of which achieved complete survival. Future studies incorporating higher challenge doses would enable a more stringent discrimination of vaccine efficacy.

A comprehensive evaluation of immune responses benefits from understanding the dose–response relationships of an adjuvant. Nevertheless, we used only an empirical fixed dose of 8.3 μg per mouse for adjuvant evaluation [20], which may have overlooked differences in immune response intensity or even side effects that could occur with different doses of the same adjuvant [39]. Future studies are warranted to include dose–response experiments to fully evaluate the dose–effect relationship and safety window of GM-CSF-Flt3L as an adjuvant.

Several additional limitations should be acknowledged. First, the present study was conducted exclusively in mice. Although mouse DC subsets such as CD103^+^ migDC are functionally analogous to human CD141^+^ DCs [40], their regulation by Flt3L and GM-CSF may differ in humans, and the immunogenicity of DNA vaccines is often lower in non-human primates and humans than in mice. Therefore, the efficacy of the GM-CSF-Flt3L adjuvant should be validated in larger animal models such as ferrets and, ultimately, in clinical settings [41]. Second, GM-CSF has been implicated in chronic inflammatory diseases and autoimmunity in certain contexts [42]. Although we did not observe overt adverse events in this acute vaccination study (Figure S1), we did not assess anti-GM-CSF autoantibody responses. Notably, a Phase I trial of an HIV peptide vaccine adjuvanted with GM-CSF found no adverse clinical or hematologic sequelae despite the development of neutralizing anti-GM-CSF antibodies in >60% of participants [43]. Nonetheless, in view of the chronic inflammatory potential of GM-CSF, the long-term safety of repeated or high-dose GM-CSF–Flt3L administration should be rigorously evaluated in future toxicology studies.

## 5. Conclusions

In summary, the use of the GM-CSF-Flt3L fusion construct as an adjuvant for an influenza DNA vaccine promotes the expansion and activation of DCs in draining lymph nodes, thereby enhancing TFH and GCB immune responses. This effect facilitates the induction of high-level specific antibodies and increases HI antibody titers, thus effectively protecting against influenza virus infection, reducing lung viral load, and ameliorating pathological damage. In the future, GM-CSF-Flt3L has the potential to serve as a universal adjuvant in various infectious diseases and cancer immunotherapies.

## 6. Patents

The fusion gene and its uses described in this manuscript are the subject of a pending Chinese patent application (No. 2026103252971) with inventors B.W., H.L., and Y.Z.

## Data Availability

All datasets generated for this study are included in the article/Supplementary Materials.

## Supplementary Materials

The following supporting information can be downloaded at https://www.mdpi.com/article/10.3390/vaccines14080671/s1: Figure S1: DNA vaccines are considered safe. Figure S2: Gating strategy and antibodies for BMDC surface activation markers. Figure S3: Gating strategies and antibodies for mouse lymph node DC activation markers (a) and DC subsets (b). Figure S4: Gating strategy and antibodies for KikGR mouse lymph node DCs. Figure S5: Gating strategies and antibodies for mouse lymph node or spleen GCB (a) and TFH (b) cells. Figure S6: Gating strategy and antibodies for T cell cytokine secretion.

## Abbreviations

The following abbreviations are used in this manuscript:

GM-CSF	Granulocyte-macrophage colony-stimulating factor
Flt3L	Fms-related tyrosine kinase 3 ligand
DCs	Dendritic cells
BMDCs	Bone marrow-derived dendritic cells
HA	Hemagglutinin
TFH	T follicular helper
GCB	Germinal center B
HCV	Hepatitis C virus
CTLs	Cytotoxic T lymphocytes
SPF	Specific pathogen free
HCD	Higher energy collisional dissociation
NCE	Normalized collision energy
LPS	Lipopolysaccharide
resDC	Resident DCs
migDC	Migratory DCs
