# Supplementary material for: A GM-CSF-Flt3L Fused Adjuvant Enhances Immune Responses and Protective Efficacy of Influenza DNA Vaccine in Mice
Source: Vaccines (Basel). 2026 Aug 2;14(8):671. doi: 10.3390/vaccines14080671 (PMC13517619; doi:10.3390/vaccines14080671)
Supplement: Supplementary file 1 [file vaccines-14-00671-s001.zip › vaccines-4447419-supplementary.pdf]

Supplementary Figures

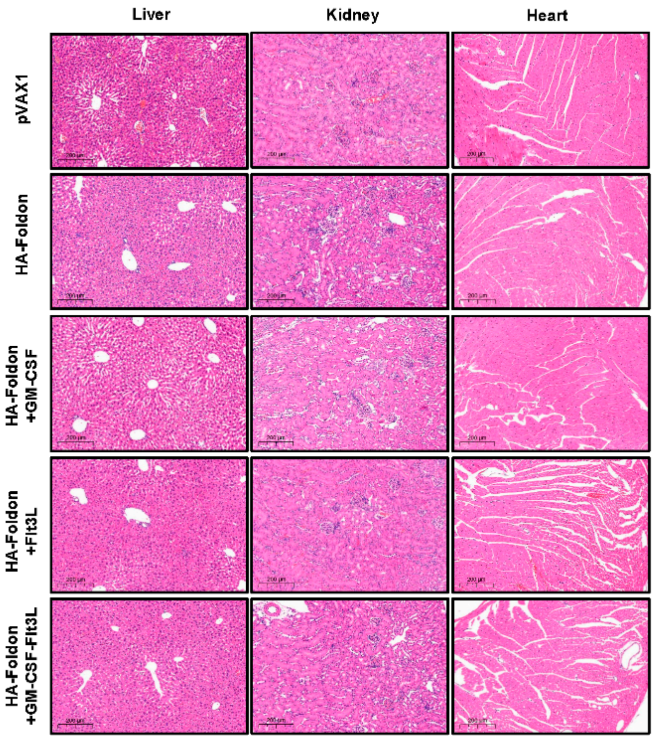

Figure S1. DNA vaccines are considered safe.
Liver, kidney, and heart tissues were collected at 2 weeks after the second immunization for H&E staining and histopathological analysis. Representative sections from each group. Scale bar, 200 μm.

Figure S2

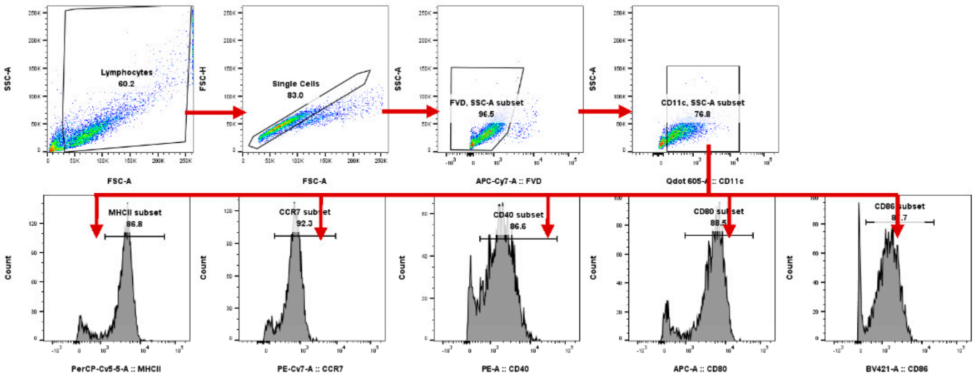

Antigen	Fluorochrome	Clone	Manufacturer	Catalog number
FVD	eFluor780		Invitrogen	65086518
MHCII	Percpcy5.5	M5/114.15.2	Biolegend	107626
CD11c	BV605	N418	Biolegend	117334
CD40	PE	3/32	Biolegend	124609
CD80	APC	16-10A1	Biolegend	104713
CCR7	PE-Cy7	4B12	Invitrogen	120123
CD86	BV421	GL-1	Biolegend	105031

Figure S2. Gating strategy and antibodies for BMDC surface activation markers.

Figure S3

a

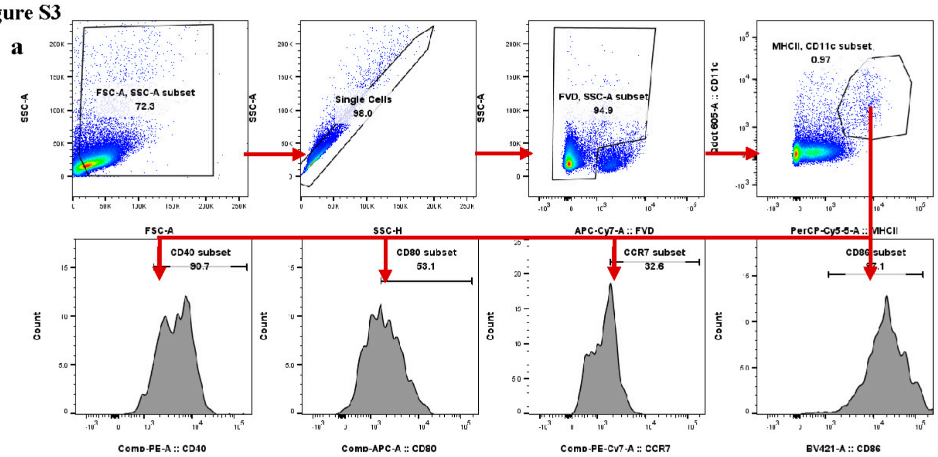

Antigen	Fluorochrome	Clone	Manufacturer	Catalog number
FVD	eFluor780		Invitrogen	65086518
MHCII	Percpcy5.5	M5/114.15.2	Biolegend	107626
CD11c	BV605	N418	Biolegend	117334
CD40	PE	3/32	Biolegend	124609
CD80	APC	16-10A1	Biolegend	104713
CCR7	PE-Cy7	4B12	Invitrogen	120123
CD86	BV421	GL-1	Biolegend	105031

b

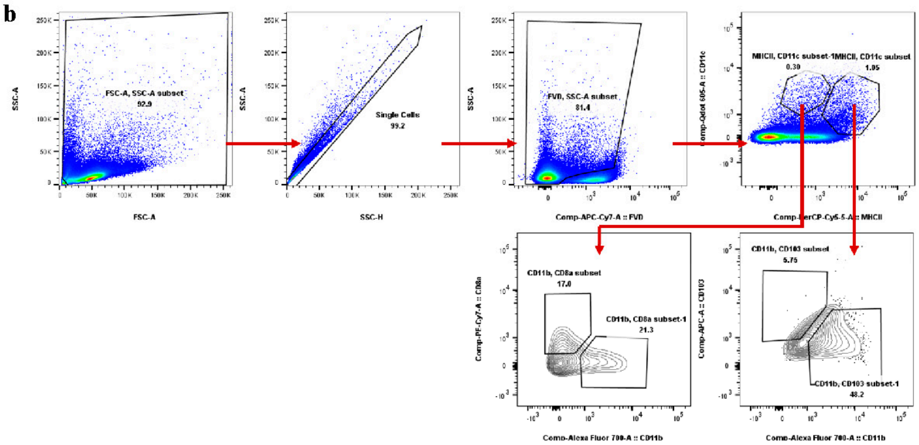

Antigen	Fluorochrome	Clone	Manufacturer	Catalog number
FVD	eFluor780		Invitrogen	65086518
MHCII	Percpcy5.5	M5/114.15.2	Biolegend	107626
CD11c	BV605	N418	Biolegend	117334
CD11b	Alexa Fluor700	M1/70	Biolegend	101222
CD8a	PE-Cy7	53-6.7	Biolegend	100722
CD103	APC	2 E7	Invitrogen	17-1031-82
CD86	BV421	GL-1	Biolegend	105031

Figure S3. Gating strategies and antibodies for mouse lymph node DC activation markers (a) and DC subsets (b).

Figure S4

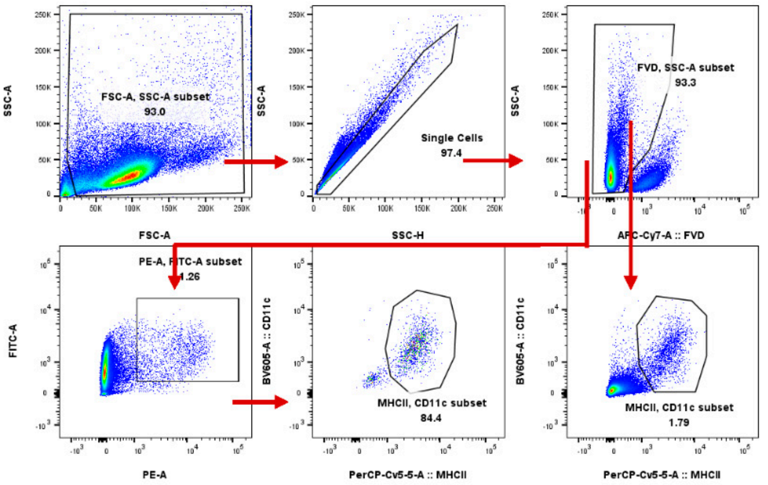

Antigen	Fluorochrome	Clone	Manufacturer	Catalog number
FVD	eFluor780		Invitrogen	65086518
MHCII	Percpcy5.5	M5/114.15.2	Biolegend	107626
CD11c	BV605	N418	Biolegend	117334

Figure S4. Gating strategy and antibodies for KikGR mouse lymph node DCs.

Figure S5

a

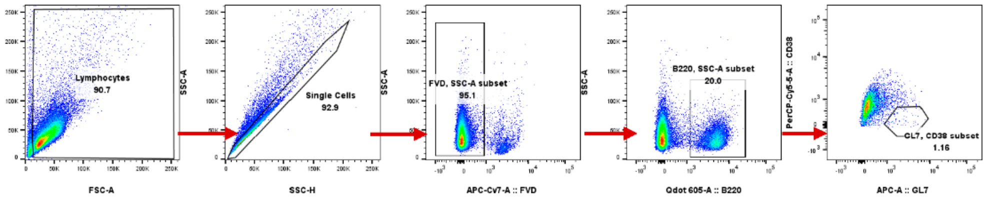

Antigen	Fluorochrome	Clone	Manufacturer	Catalog number
FVD	eFluor780		Invitrogen	65086518
B220	BV605	RA3-6B2	Biolegend	103243
CD38	Percp-eFluor710	90	Invitrogen	46038182
GL7	APC	GL7	Biolegend	144617

b

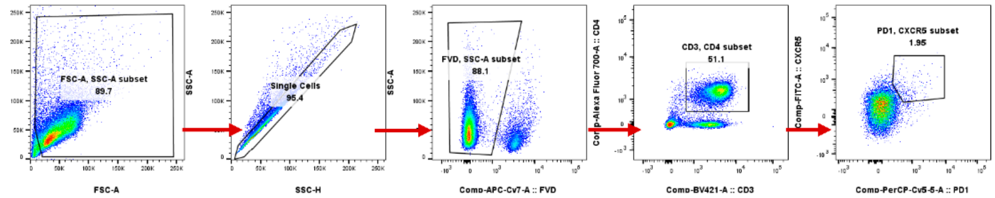

Antigen	Fluorochrome	Clone	Manufacturer	Catalog number
FVD	eFluor780		Invitrogen	65086518
CD3	BV421	17A2	Biolegend	100228
CD4	Alexa Fluor700	GK1.5	Biolegend	100430
PD-1	Percpcy5.5	29F.1A12	Biolegend	135207
CXCR5	FITC	L138D7	Biolegend	145519

Figure S5. Gating strategies and antibodies for mouse lymph node or spleen GCB (a) and TFH (b) cells.

Figure S6

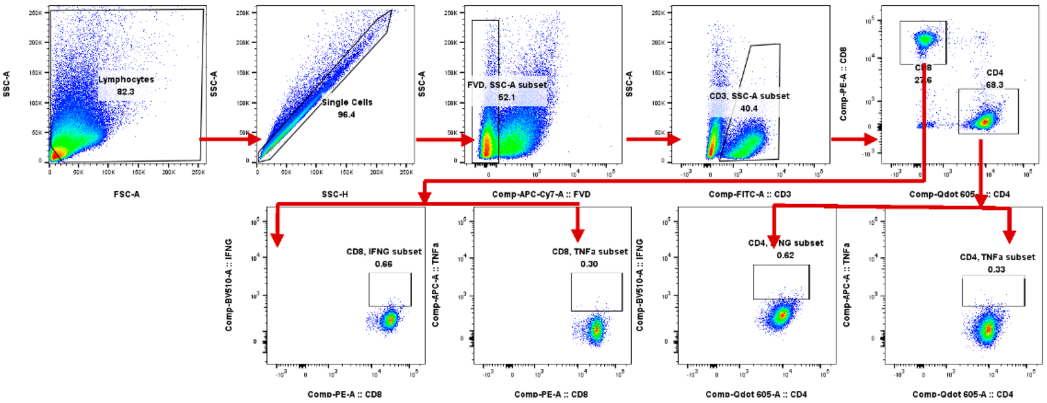

Antigen	Fluorochrome	Clone	Manufacturer	Catalog number
**T cell surface staining**				
FVD	eFluor780		Invitrogen	65086518
CD3	FITC	145-2C11	Biolegend	100306
CD4	BV605	GK1.5	Biolegend	100451
CD8a	PE	53-6.7	Biolegend	100707
**T cell intracellular cytokine staining**				
IFN-g	BV510	XMG1.2	Biolegend	505841
TNF-a	APC	MP6-XT22	Biolegend	506307

Figure S6. Gating strategy and antibodies for T cell cytokine secretion.
